# Clinical Implications of Upregulated *RSAD2* Gene Expression in Hepatocellular Carcinoma

**DOI:** 10.3390/diseases13120395

**Published:** 2025-12-08

**Authors:** Leung Li, Nelson L. S. Tang, Stephen L. Chan, David Ryan Johnson, Frankie Mo, Jane Koh, Tsz-Ki Kwan, Edwin P. Hui, Landon Long Chan, Kit F. Lee, Simon Chun Ho Yu, Winnie Yeo

**Affiliations:** 1 State Key Laboratory of Translational Oncology, Department of Clinical Oncology, Prince of Wales Hospital, Faculty of Medicine, Hong Kong Cancer Institute, The Chinese University of Hong Kong, Hong Kong SAR, China; 2 Department of Chemical Pathology, Prince of Wales Hospital, Faculty of Medicine, Li Ka Shing Institute of Health Sciences, The Chinese University of Hong Kong, Hong Kong SAR, China; 3 Hong Kong Branch of CAS Center for Excellence in Animal Evolution and Genetics and KIZ/CUHK Joint Laboratory of Bioresources and Molecular Research in Common Diseases, Hong Kong SAR, China; 4 Functional Genomics and Biostatistical Computing Laboratory, CUHK Shenzhen Research Institute, Shenzhen 518000, China; 5 Cytomics Limited, Hong Kong Science Park, Hong Kong SAR, China; 6 Department of Surgery, Prince of Wales Hospital, Shatin, Faculty of Medicine, The Chinese University of Hong Kong, Hong Kong SAR, China; 7 Department of Diagnostic and Interventional Radiology, Prince of Wales Hospital, Faculty of Medicine, The Chinese University of Hong Kong, Hong Kong SAR, China

**Keywords:** viperin, liver cancer, quantitative real-time polymerase chain reaction, quality of life, messenger ribonucleic acid

## Abstract

**Background:** *JAK/STAT* interferon signaling interacts with the *PI3K/AKT/mTOR* pathway to drive hepatocellular carcinoma (HCC) progression and metastasis. *RSAD2*, an interferon-inducible gene, is upregulated by the *PI3K/AKT/mTOR* pathway and serves as a key factor for metabolic reprogramming to promote stem-like properties of cancer stem cells and tumor proliferation. In patients with resected HCC, *RSAD2* upregulation showed an association with microvascular invasion, which is a proven risk factor for developing HCC metastasis. This clinical observation was compatible with preclinical findings. On the other hand, *RSAD2* upregulation has been reported to confer poor prognosis in breast and gastric cancers. However, further clinical study of *RSAD2* in HCC is lacking. As a result, we investigated the clinical implications of *RSAD2* gene expression in HCC patients, in terms of its associations with survival, the presence of extra-hepatic metastasis, and other clinical manifestations. **Methods:** We studied 309 treatment-naïve HCC patients, as well as data from the TCGA and GTEx databases. **Results:** *RSAD2* gene expression was differentially upregulated in HCC tumors when compared to normal liver tissues (*p* < 0.01). Elevated *RSAD2* mRNA levels in the blood and the presence of extra-hepatic metastasis were independent prognostic factors for poor overall survival (OS) (*p* < 0.01). The median OS of patients with high *RSAD2* expression vs. low expression were 5.4 vs. 14.2 months, respectively (*p* < 0.01). A high *RSAD2* mRNA level was significantly correlated with the presence of extra-hepatic metastasis, nutritional disturbance, and functional impairment after controlling for confounding clinical factors (*p* < 0.05). **Conclusions:** High *RSAD2* gene expression is associated with poorer OS, the presence of extra-hepatic metastasis, and quality-of-life disturbances in HCC patients.

## Simple Summary

*RSAD2*, an interferon-inducible gene and an emerging biomarker in cancer, was found to drive tumor progression in preclinical studies. A clinical study showed supportive evidence that, in resected hepatocellular carcinoma (HCC), *RSAD2* gene upregulation had an association with blood vessel invasion, which is a proven risk factor for developing metastasis. However, clinical research on *RSAD2* in HCC is lacking, and it is of clinical interest whether *RSAD2* has an association with metastatic disease. Furthermore, *RSAD2* gene upregulation has been reported to be associated with poor survival in patients with breast and stomach cancer, but its prognostic value in HCC has not been formally evaluated. This study involved 309 HCC patients and investigated the clinical implications of *RSAD2* gene upregulation in peripheral blood, in terms of its associations with survival, the presence of metastasis, and other clinical manifestations. Higher *RSAD2* expression in the blood was significantly associated with poorer survival and was associated with the presence of metastasis. Moreover, HCC patients with increased *RSAD2* expression were more likely to have nutritional disturbance, appetite loss, fatigue, and functional impairment.

## 1. Introduction

Hepatocellular carcinoma (HCC) accounts for the sixth most common cancer and the third leading cause of cancer death in the world [1]. The majority of the world’s HCC cases are diagnosed in China [2].

Inflammation, immune system activation, and immune evasion play key roles in HCC development and progression [3]. In particular, the interferon response in HCC has been shown to induce the metabolic reprogramming of tumor cells, the tumor micro-environment, and immune cells to facilitate HCC progression and metastasis [4]. The interferon response has also been shown to interact with *PI3K/AKT/mTOR* signaling to drive HCC proliferation and tumor cell migration [5]. The interferon response gene *RSAD2*, upregulated mechanistically by the *JAK/STAT* interferon response and *PI3K/AKT/mTOR* pathways, is a key factor for metabolic reprogramming to enhance lipogenesis and glycolysis, thereby promoting stem-like properties of cancer stem cells and tumor proliferation [6]. *RSAD2* knockdown in cancer cell lines was shown to suppress tumor progression and reverse metabolic reprogramming, implicating it as a driver of tumor proliferation [7].

However, the current literature indicates a lack of clinical studies on *RSAD2* in HCC patients to validate these pre-clinical mechanistic findings. In a report on patients with resected HCC, *RSAD2* upregulation in tumors was linked to microvascular invasion, which is a proven risk factor for developing HCC metastasis [8,9]. Microvascular invasion in resected HCC has been used to identify high-risk patients who will develop extra-hepatic metastases [9]. Moreover, Bertuzzo et al. reported that microvascular invasion and a cell-count-based inflammatory marker were the strongest risk factors for HCC recurrence [10]. This calls for an investigation on the clinical implications of *RSAD2* gene expression in HCC patients—whether *RSAD2* upregulation is associated with the presence of metastasis and poorer survival.

Studies evaluating *RSAD2* in other cancers commonly reported its prognostic value with mixed findings. In particular, *RSAD2* was reported to be an adverse prognostic factor in breast and gastric cancers but a favorable prognostic factor in oral cancer and melanoma [7,11,12,13,14]. Prognostic studies of *RSAD2* in cancer were mainly genomic analyses using public databases. A caveat concerning interpreting online database studies is that clinical factors provided by these databases may not be adequate to support post hoc prognostic analyses, thereby compromising the accuracy of prognostic models. Dedicated prognostic studies of *RSAD2* with relevant and comprehensive clinical data from patients with cancer are lacking.

As a result, we set out to investigate the clinical significance of *RSAD2* gene expression in an HCC patient cohort. In the first part of the study, we evaluated whether *RSAD2* was differentially expressed in HCC tumor samples compared to normal tissues using genomic repositories. The second part of this study involved a prospective cohort of treatment-naïve HCC patients. We determined their peripheral blood leucocytic *RSAD2* mRNA transcript levels and then evaluated their clinical implications in terms of prognostic value and associations with clinical factors (including extra-hepatic metastasis) and patients’ symptoms as captured by patient-reported outcome measures.

## 2. Materials and Methods

### 2.1. RSAD2 Gene Expression in HCC

To explore whether *RSAD2* is differentially expressed in HCC compared to normal liver tissues, we analyzed the combined database of The Cancer Genome Analysis (TCGA) (https://www.cancer.gov/tcga, accessed on 26 April 2025) and Genotype-Tissue Expression (GTEx) (https://gtexportal.org, accessed on 26 April 2025), in which the batch effect was minimized after sample re-analyses using a uniform RNA sequencing (RNA-seq) pipeline. Data were analyzed using the Xena multi-omic exploration tool, University of California, Santa Cruz, CA, USA [15]. *RSAD2* gene expressions in HCC samples (the database of Genotypes and Phenotypes [dbGaP] accession number phs000178.v11.p8) were compared to normal liver tissue samples (dbGaP accession number phs000424.v10.p2) using Welch’s *t*-test.

### 2.2. Local HCC Patient Cohort

This study was approved by the Joint Chinese University of Hong Kong—New Territories East Cluster Clinical Research Ethics Committee of Prince of Wales Hospital, Hong Kong on 13 October 2006 (reference number CRE-2006.340). Newly diagnosed HCC patients were prospectively recruited. Diagnosis was based on either tumor biopsy or a combination of typical dynamic imaging findings and elevated α-fetoprotein (AFP). Patients were eligible for the study if they were treatment-naïve. Major exclusion criteria included cognitive impairment or a history of another malignancy. Written informed consent was obtained from every patient.

### 2.3. Demographic, Clinical, and Laboratory Data

Baseline demographic and clinical data were collected. On the day of consent, blood tests were taken for cell counts, liver and renal biochemistries, coagulation profile, AFP, hepatitis serology, and *RSAD2* mRNA level.

### 2.4. Peripheral Blood RSAD2 Gene Expression Quantification

#### 2.4.1. Isolation of Peripheral Blood Leucocytes and Extraction of Total RNA

Peripheral blood in ethylenediaminetetraacetic acid (EDTA) was used for *RSAD2* expression analysis. Peripheral blood mononuclear cells (PBMCs) and granulocytes were isolated by density gradient centrifugation using Lymphoprep™ (Serumwerk Bernburg AG, Bernburg, Germany). Total RNA from leucocytes and whole blood were extracted via a modified acid guanidinium thiocyanate–phenol–chloroform (AGPC) protocol suggested by the manufacturer (Molecular Research Center, Inc., OH, USA) [16]. Then, 250 μL of the TRI Reagent^®^ LS (TB126) was added to the PBMCs and granulocytes, while 300 µL of the TRI Reagent^®^ BD (TB120) was added to the whole blood. 1-Bromo-3-chloropropane (B9673, Sigma-Aldrich, MA, USA) was used for phase separation. Subsequent washing and elution were performed by isopropanol (I9516, Sigma-Aldrich, MA, USA) and ethanol (4116-4104, Daejung, Siheung-si, South Korea).

#### 2.4.2. cDNA Synthesis

First-strand cDNA was reverse transcribed from the total RNA using the PrimeScript RT Reagent Kit (#RR037A, Takara Bio, Shiga, Japan). The 10 µL reverse transcription system was used, including 2 µL of 5X PrimeScript Buffer (for real-time), 0.5 µL PrimeScript RT Enzyme Mix I, 0.5 µL Oligo dT Primer (50 µM), 2 µL Random 6mers (100 µM), and 5 µL of RNA (up to 1 µg/µL) from the samples. Reverse transcription was performed in the C1000 Touch Thermal Cycler (Bio-Rad, Hercules, CA, USA) under the following conditions: sample incubation at 37 °C for 15 min, followed by enzyme denaturation at 85 °C for 5 s, and then storage at 4 °C for short-term preservation before refrigeration storage.

#### 2.4.3. RSAD2 Gene Expression Quantification

The synthesized cDNA was diluted and duplicated to optimize the real-time qPCR performance. In each batch of the qPCR experiment, the same reference sample was used, and a serial 2-fold dilution was carried out to establish PCR efficiency. The TB Green^®^ Premix Ex Taq™ II (Tli RNase H Plus) kit (#RR820A, Takara Bio, Shiga, Japan) and *RSAD2* gene primers (see Table 1) were used in the reaction, performed in an LC480 thermal cycler (Roche, Basel, Switzerland). The qPCR conditions included a pre-incubation step at 95 °C for 30 s, followed by 45 cycles of amplification (95 °C for 5 s, 55 °C for 30 s, and 72 °C for 20 s), a melting phase (95 °C for 1 min, 40 °C for 1 min and 65 °C for 20 s), and a cooling step at 40 °C for 30 s. Patients’ *RSAD2* mRNA transcript levels were expressed as fold change (FC) against the same normal healthy donor as the calibrator, using the amplification efficiency-corrected delta delta cycle threshold (ddCT) method [17].

FC = eff *RSAD2* ^ (Ct*RSAD2*_calibrator − Ct*RSAD2*_mean) ÷ eff *UBC* ^ (Ct*UBC*_calibrator − Ct*UBC*_mean)]

### 2.5. Health-Related Quality of Life (HRQoL) Assessment

Patients filled in the European Organization for Research and Treatment of Cancer (EORTC) QLQ-C30 and QLQ-HCC18 questionnaires at study entry [18,19]. C30 and HCC18 index scores were calculated [20].

### 2.6. Follow-Up

Patients were followed up until death.

### 2.7. Sample Size Estimation and Missing Data Handling

The median overall survival (OS) was estimated to be around 6 months from prior local data [21]. Assuming a hazard ratio (HR) of 2.0 for high *RSAD2* expression with two-sided alpha at 0.05 and 80% power, we aimed to recruit 298 patients. There was no prior observation for the novel variable of peripheral blood *RSAD2* mRNA levels to guide reasonable imputation for missing data. Furthermore, in order to calculate C30 and HCC18 index scores, all scores from QLQ-C30 and QLQ-HCC18, respectively, had to be available. As a result, we adopted the complete-case analysis approach.

### 2.8. Statistical Analyses

Baseline *RSAD2* mRNA transcript levels and clinical and HRQoL factors were analyzed by standard descriptive tests. OS was defined as the time between the date of recruitment and the date of death. Patients alive or lost to follow-up were censored at the date of last follow-up. Kaplan–Meier analysis was used for survival estimates. A log-rank test was used to compare survival distributions. Cox proportional hazards models were used to identify non-overlapping independent prognostic factors among clinical and HRQoL data for OS. Two-tailed *p*-values of less than 0.05 were considered statistically significant. Correlations between *RSAD2* mRNA levels and HRQoL factors were analyzed using Student’s *t*-test, Wilcoxon rank-sum test (parametric and non-parametric tests were conducted as sensitivity analyses to account for different distributional assumptions of the novel *RSAD2* variable), and univariate logistic regressions. Multivariate logistic regressions were used to control for demographic and clinical factors. Data were analyzed using SAS version 9.4 (SAS institute, Cary, NC, USA). Study methods and results are reported in accordance with the Strengthening the Reporting of Observational Studies in Epidemiology (STROBE) Statement [22].

## 3. Results

### 3.1. RSAD2 Gene Expression in HCC Tumors

**Figure 1:**
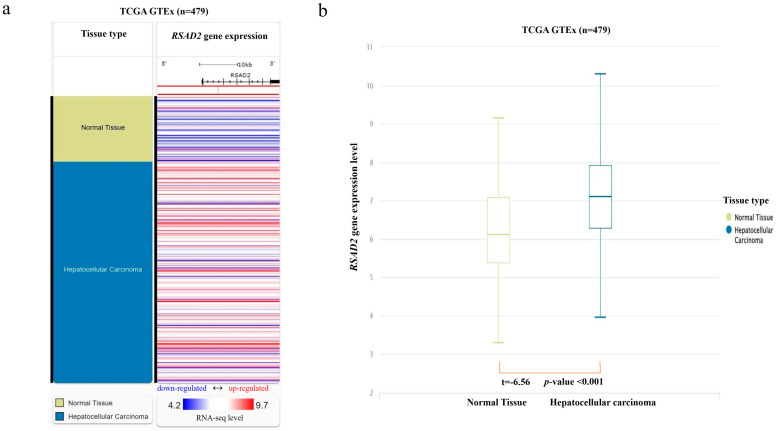
*RSAD2* mRNA expression in the combined TCGA and GTEx databases. (**a**) Heatmap of *RSAD2* mRNA expression in HCC tumors vs. normal liver tissue samples. Each horizontal bar represents an individual patient’s *RSAD2* mRNA gene expression level, and a color gradient is applied according to the RNA-seq level (red representing upregulated mRNA expression; white representing average expression; and blue representing downregulated expression). (**b**) Boxplot of *RSAD2* mRNA expression in HCC compared to normal liver. mRNA expression level was quantified according to the RNA-Seq by Expectation–Maximization algorithm.

From the combined TCGA and GTEx database, RNA-seq data were found in 369 HCC and 110 normal liver tissue samples. *RSAD2* mRNA transcript abundance in HCC tumors was compared against matching controls. The results showed that *RSAD2* gene expression was significantly higher in HCC tumors when compared to normal liver tissues (*p* < 0.01) (shown in Figure 1).

### 3.2. Characteristics of the HCC Patient Cohort

From 2009 to 2017, 309 HCC patients had complete data for analysis (among the 340 recruited patients, 11 patients’ *RSAD2* mRNA levels could not be determined, and 20 patients had incomplete HRQoL data). Table 2 shows their baseline clinical characteristics. The median age at diagnosis was 59 (range 27–86) years. Eighty-seven percent were male. Most patients (80%) had evidence of hepatitis B infection. Focal, multifocal, diffuse liver tumors were found in 98 (32%), 90 (29%), and 121 (39%) patients, respectively. Seventy-one (23%) patients had extra-hepatic metastasis (AJCC 8th edition stage IV) [23]. The median follow-up duration was 116 (95% confidence interval [CI] 87–120) months. The median OS was 9.5 (95% CI 6.1–14.3) months. Supplementary Table S1 shows their baseline HRQoL scores in QLQ-C30 and QLQ-HCC18. The mean ‘global health status’ score was 53.4 (standard deviation [SD] 25.1).

### 3.3. Peripheral Blood RSAD2 mRNA Transcript Level Analyses

**Figure 2:**
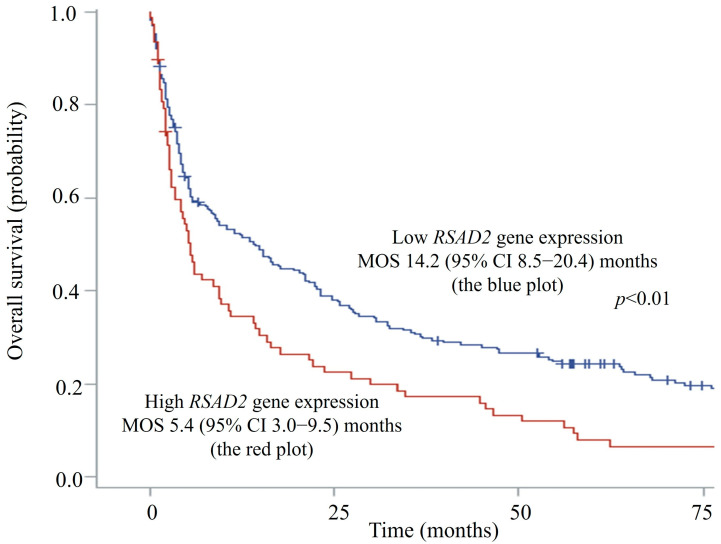
Overall survival plots of hepatocellular carcinoma patients with high vs. low *RSAD2* mRNA expression. CI—confidence interval; MOS—median overall survival.

The median *RSAD2* mRNA transcript level in patients’ circulating leucocytes was 1.16 (interquartile range 0.33–2.89) FC. Since *RSAD2* gene expression demonstrated an asymmetrical skewed distribution, expression levels of more than the 75th percentile (2.89 FC) were ranked as high to facilitate risk of death analyses using Cox models. Seventy-six patients had high *RSAD2* gene expression in peripheral blood, whereas 233 patients had low expression. The median OS was significantly shorter in patients with high *RSAD2* gene expression when compared to those with low *RSAD2* expression, 5.4 (95% CI, 3.0–9.5) vs. 14.2 (95% CI, 8.5–20.4) months, respectively (log-rank *p* < 0.01). Figure 2 shows the Kaplan–Meier survival curves of patients with respect to *RSAD2* gene expressions.

### 3.4. Univariate Prognostic Analyses of RSAD2 mRNA Level and Clinical Factors

Table 2 shows the results of univariate Cox proportional hazards analyses of clinical factors. Higher *RSAD2* mRNA levels, the presence of extra-hepatic metastasis, a higher white cell count (WCC), lower hemoglobin levels, and higher alanine transferase were significantly associated with poorer OS (*p* < 0.01). Patients with poor liver function (Child–Pugh classes B or C) had significantly poorer OS than patients with preserved liver function (class A) (*p* < 0.01). Patients with more advanced HCC (Barcelona Clinic Liver Cancer stage B, C or D) had significantly poorer OS than patients with earlier disease (stage A) (*p* < 0.001) [24].

### 3.5. Multivariate Analyses for Independent Prognostic Factors

The results of multivariate Cox proportional hazards models with *RSAD2* gene expression and non-overlapping clinical and HRQoL factors are shown in Table 3. The presence of extra-hepatic metastasis, high *RSAD2* mRNA gene expression, higher WCC, and anemia were independent poor prognostic factors for OS (*p* < 0.01). When AFP was included in the multivariate Cox model, the *RSAD2* mRNA level was no longer identified as a significant factor. In other words, AFP was a stronger prognostic factor than *RSAD2*.

### 3.6. Correlates of RSAD2 mRNA Levels and Extra-Hepatic Metastases, as Well as Other Clinical Manifestations

Higher *RSAD2* gene expression was significantly correlated with the presence of extra-hepatic metastasis (*p* < 0.03). However, *RSAD2* levels had no significant association with age, sex, cell counts, hepatitis B, hepatitis C, or liver or renal function. Regarding other clinical manifestations, higher *RSAD2* gene expression in circulating leucocytes were associated with worse symptoms in ‘appetite loss’, ‘fatigue’, ‘nutritional concern’, and ‘body image’ (*p* < 0.03) (see Table 4).

### 3.7. Multivariate Correlation Models Between RSAD2 mRNA Transcript Levels and Extra-Hepatic Metastases, as Well as Other Clinical Manifestations

After controlling for potentially confounding clinical factors in multivariate logistic regressions, extra-hepatic metastasis remained significantly correlated with upregulated *RSAD2* gene expression (OR, 2.94; 95% CI, 1.29–6.70; *p* < 0.03) (Table 5). The presence of extra-hepatic metastasis was among the factors that best predicted a higher *RSAD2* transcript level in peripheral blood.

## 4. Discussion

From the current study, *RSAD2* gene expression was found to be heightened in HCC patients’ tumor tissues and/or peripheral blood leucocytes. *RSAD2* gene expression level and extra-hepatic metastasis were both identified as independent prognostic factors for OS in HCC. Patients with a high *RSAD2* mRNA transcript level had significantly shorter median OS than those with a low *RSAD2* level (5 vs. 14 months, respectively). Although its prognostic value was not superior to AFP, further research is needed to evaluate whether integrating *RSAD2* can improve HCC staging systems. For instance, various staging systems have been incorporated with non-anatomical prognostic biomarkers [25,26].

A high *RSAD2* mRNA level was significantly correlated with the presence of extra-hepatic metastasis. Multivariate correlation analyses were performed after controlling for potentially confounding factors, including age, sex, cell counts, hepatitis B/C, and liver or renal function. Since treatment-naïve patients were recruited, therapy-related alteration in *RSAD2* levels could be avoided.

The association between *RSAD2* and extra-metastasis is compatible with the current mechanistic knowledge of *RSAD2* in cancers. Clinical reports have also shown an association between inflammatory status and the development of metastasis in melanoma and breast cancer [27,28]. Microvascular invasion, a known risk factor for developing metastasis, was more frequently observed in HCC patients with *RSAD2* upregulation [8,9]. These findings are compatible with the results of our current study.

To date, AFP has remained the most widely used tumor marker for HCC detection. However, 30–40% of HCC patients had negative AFP [29]. Even among patients with elevated AFP, the level cannot differentiate between the presence or absence of extra-hepatic spread. Therefore, there has been a clinical need for a biomarker to indicate the presence of HCC metastasis. From the multivariate regressions between *RSAD2* gene expression, adjusting for clinical factors, the presence of extrahepatic metastasis was among the factors that best predicted a higher *RSAD2* gene expression level. Based on the findings of our study, *RSAD2* upregulation in peripheral blood would suggest a need for systemic imaging to search for extra-hepatic metastasis.

This study used HRQoL instruments to measure and quantify patients’ symptoms into analyzable data, in order to examine the relationships between *RSAD2* gene upregulation and patients’ clinical manifestations. Remarkably, peripheral blood *RSAD2* gene upregulation (signifying inflammatory cascade activation) demonstrated significant correlations with appetite loss, nutritional concern, fatigue, and social functional impairment. These results are logical since such clinical manifestations are typically found in patients in an inflammatory state and with constitutional symptoms [30]. Chiba et al. demonstrated a correlation between pro-inflammatory state and cancer cachexia in an animal model [31]. This observation also substantiated our current clinical findings.

We acknowledge certain limitations in this study. Firstly, it is unclear whether the current results are generalizable to patients with other cancers. Further studies are warranted. Secondly, this study provided valuable observations in Chinese patients, but it is unknown if the findings are applicable to HCC patients of other ethnicities. Future validation in a multi-ethnic cohort is indicated.

## 5. Conclusions

*RSAD2* gene expression was differentially upregulated in HCC tumors when compared to normal liver tissues. Increased *RSAD2* gene expression in peripheral blood and extra-hepatic metastasis are significant independent prognostic factors for OS in HCC. An elevated *RSAD2* mRNA transcript level in circulating leucocytes was significantly correlated with the presence of extra-hepatic metastasis, and it was associated with clinical features that were suggestive of constitutional symptoms (nutritional concern, appetite loss, fatigue, and functional impairment). An elevated peripheral blood *RSAD2* level could suggest a search for extra-hepatic metastasis. Further research is needed to examine the clinical significance of *RSAD2* gene expression in other cancer types.

## Figures and Tables

**Table 1:** Primer sequences used for quantification of *RSAD2* mRNA transcript levels in peripheral blood.

Primer	Primer Sequence
*RSAD2* gene (forward) primer sequence	5′-CGACGGTGCGAGAATACCTGGGCAAGTTGG-3′
*RSAD2* gene (reverse) primer sequence	5′-CCAGCGTCCCGTCACAGGAGATAGCGAGAATG-3′
Ubiquitin C gene (forward) primer sequence	5′-CGTCCGTCGCCAGCCGGGATTTGGGTCG-3′
Ubiquitin C gene (reverse) primer sequence	5′-CGACGCAGCCCACGAAGATCTGCATTGTCAAGT-3′

**Table 2:** Demographic and clinical characteristics of the patients.

Clinical Factors	Number	%	HR	95% CI	*p*-Value
Age ≤ 65 years	215	69.6	0.919	0.708–1.193	0.5248
Male gender	269	87.1	1.191	0.826–1.717	0.3482
Hepatitis B	248	80.3	0.944	0.702–1.270	0.7035
Hepatitis C	23	7.4	1.149	0.748–1.765	0.5253
Hemoglobin ≤ 10 g/dL	17	5.5	2.064	1.259–3.384	0.0041
White cell count > 10 × 10^9^/L	38	12.3	2.856	1.998–4.082	<0.0001
Platelet count < 100 × 10^9^/L	47	15.2	0.643	0.455–0.909	0.0125
International normalized ratio > 1.4	14	4.5	1.667	0.933–2.979	0.0843
Bilirubin ≥ 20 μmol/L	138	44.7	1.953	1.529–2.496	<0.0001
Albumin ≤ 35 g/L	102	33.0	2.353	1.819–3.043	<0.0001
Alanine aminotransferase > 2 × ULN	46	14.9	1.569	1.118–2.201	0.0091
Alpha feto-protein ≥ 200 ng/mL	140	45.3	2.421	1.888–3.104	<0.0001
*RSAD2* mRNA level elevated	76	24.6	1.490	1.130–1.965	<0.0001
Child–Pugh class					
A	224	72.5	1.000	-	-
B	75	24.3	2.145	1.624–2.833	<0.0001
C	10	3.2	10.006	5.129–19.520	<0.0001
BCLC					
A	57	18.5	1.000	-	-
B	58	18.8	3.206	2.013–5.106	<0.0001
C	157	50.8	5.827	3.847–8.827	<0.0001
D	37	11.9	20.633	12.149–35.044	<0.0001
Tumor Morphology					
Uninodular	98	31.7	1.000		
Multinodular	90	29.1	2.967	2.118–4.157	<0.0001
Diffuse	121	39.2	3.195	2.459–4.152	<0.0001
AJCC Stage					
I/II/III	238	77	1.000	-	-
IV (extra-hepatic metastasis)	71	23	4.696	3.455–6.383	<0.0001

Legends: AJCC Stage—the American Joint Committee on Cancer staging system 8th edition; BCLC—the Barcelona Clinic Liver Cancer staging system; CI—confidence interval; HR—hazard ratio; mRNA—messenger ribonucleic acid; ULN—upper limit of normal; ×—multiple(s) of.

**Table 3:** Multivariate Cox proportional hazards analyses of prognostic factors for overall survival.

	Continuous HRQoL Variables					Dichotomized HRQoL Variables			Index Score			
	HR	95% CI		*p*-Value	HR	95% CI		*p*-Value	HR	95% CI		*p*-Value
EORTC QLQ-C30												
White cell count	1.104	1.061	1.149	<0.0001	1.102	1.060	1.145	<0.0001	1.112	1.070	1.157	<0.0001
Hemoglobin level	0.901	0.841	0.965	0.0027	0.888	0.830	0.950	0.0006	0.879	0.822	0.939	0.0001
Extra-hepatic metastasis	3.243	2.348	4.479	<0.0001	3.403	2.461	4.707	<0.0001	3.183	2.308	4.390	<0.0001
High *RSAD2* mRNA level	1.501	1.132	1.989	0.0047	1.509	1.137	2.001	0.0043	1.568	1.183	2.078	0.0018
Physical functioning	0.648	0.482	0.870	0.0039								
Fatigue					1.689	1.263	2.258	0.0004				
Appetite loss	1.657	1.354	2.028	<0.0001	1.716	1.258	2.342	0.0007				
C30 index score									3.089	2.214	4.311	<0.0001
EORTC QLQ-HCC18												
White cell count	1.107	1.065	1.151	<0.0001	1.104	1.063	1.147	<0.0001	1.104	1.062	1.147	<0.0001
Hemoglobin level	0.884	0.825	0.947	0.0004	0.894	0.835	0.958	0.0015	0.875	0.818	0.936	0.0001
Extra-hepatic metastasis	3.249	2.346	4.499	<0.0001	3.371	2.441	4.657	<0.0001	3.382	2.452	4.664	<0.0001
High *RSAD2* mRNA level	1.563	1.178	2.072	0.0019	1.557	1.173	2.065	0.0022	1.520	1.147	2.016	0.0036
Nutritional concern	1.889	1.359	2.626	0.0002								
Body image					1.547	1.091	2.194	0.0144				
Abdominal swelling	1.357	1.095	1.681	0.0055	1.849	1.305	2.620	0.0005				
HCC18 index score									2.918	2.033	4.190	<0.0001

Legends: CI—confidence interval; EORTC—the European Organization for Research and Treatment; HR—hazard ratio; HRQoL—health-related quality of life; mRNA—messenger ribonucleic acid.

**Table 4:** Clinical symptoms showing significant correlations with *RSAD2* mRNA transcript level.

	Low *RSAD2* mRNA Level		High *RSAD2* mRNA Level		*t*-Test	Wilcoxon	Logistic Regression			
	Mean	SD	Mean	SD	*p*-Value *	*p*-Value **	OR	95% CI		*p*-Value ***
EORTC QLQ-C30										
Emotional functioning	78.22	21.06	70.01	25.25	0.0313	0.0408	0.470	0.234	0.943	0.0335
Social functioning	81.58	26.48	65.26	30.46	0.0005	0.0002	0.352	0.191	0.649	0.0008
Global health status	62.43	23.79	51.12	24.97	0.0038	0.0036	0.389	0.202	0.749	0.0047
Fatigue	33.14	28.05	46.09	29.68	0.0052	0.0045	2.214	1.252	3.917	0.0063
Dyspnea	21.05	28.61	33.80	32.49	0.0104	0.0073	2.024	1.168	3.507	0.0122
Appetite loss	21.64	31.80	34.98	34.23	0.0120	0.0059	1.896	1.139	3.157	0.0138
Financial difficulties	42.11	34.80	55.16	37.25	0.0238	0.0237	1.629	1.063	2.497	0.0253
C30 index score	23.43	17.65	32.54	18.65	0.0018	0.0008	4.306	1.669	11.108	0.0025
EORTC QLQ-HCC18										
Fatigue	27.29	25.29	36.03	24.67	0.0259	0.0104	2.138	1.083	4.221	0.0282
Body image	19.88	23.45	28.05	22.84	0.0247	0.0061	2.328	1.102	4.917	0.0265
Nutritional concern	18.83	20.48	30.09	21.23	0.0008	<0.0001	4.349	1.778	10.641	0.0013
Fever	3.22	10.65	6.57	14.58	0.0745	0.0478	3.206	0.716	14.359	0.1277
HCC18 index score	19.62	17.82	26.05	16.89	0.0178	0.0029	3.287	1.210	8.932	0.0199

* *p*-value obtained by *t*-test, ** *p*-value obtained by Wilcoxon rank sum test, *** *p*-value obtained by logistic regression. Legends: CI—confidence interval; EORTC—the European Organization for Research and Treatment; mRNA—messenger ribonucleic acid; OR—odds ratio; SD—standard deviation.

**Table 5:** Multivariate logistic regression analyses between *RSAD2* mRNA transcript levels and extra-hepatic metastasis and other clinical manifestations for clinical factors.

(i) EORTC QLQ-C30 with clinical factors.				
	Logistic Regression			
	OR	95% CI		*p*-value
Social functioning	0.352	0.191	0.649	0.0008
(ii) C30 index score with clinical factors.				
	Logistic Regression			
	OR	95% CI		*p*-value
C30 index score	4.306	1.669	11.108	0.0025
(iii) EORTC QLQ-HCC18 with clinical factors.				
	Logistic Regression			
	OR	95% CI		*p*-value
Nutritional concern	4.349	1.778	10.641	0.0013
(iv) HCC18 index score with clinical factors.				
	Logistic Regression			
	OR	95% CI		*p*-value
Extra-hepatic metastasis	2.935	1.285	6.703	0.0106

Legends: CI—confidence interval; EORTC—the European Organization for Research and Treatment; OR—odds ratio.

## Data Availability

The datasets generated and analyzed during the current study are not publicly available due to embedded individual privacy but are available on request from the corresponding author.

## Supplementary Materials

The following supporting information can be downloaded at https://www.mdpi.com/article/10.3390/diseases13120395/s1. Table S1. Health-related quality of life characteristics of the patients.

## Abbreviations

The following abbreviations are used in this manuscript:

AFP	α-fetoprotein
AJCC	the American Joint Committee on Cancer staging system
AGPC	acid guanidinium thiocyanate–phenol–chloroform
cDNA	complementary deoxyribonucleic acid
CI	confidence interval
CRP	C-reactive protein
dbGaP	the database of Genotypes and Phenotypes
ddCT	delta delta cycle threshold
EDTA	ethylenediaminetetraacetic acid
EORTC	the European Organization for Research and Treatment of Cancer
FC	fold change
GTEx	genotype–tissue expression
HRQoL	health-related quality of life
IL	interleukin
mRNA	messenger ribonucleic acid
OS	overall survival
PBMC	peripheral blood mononuclear cell
qPCR	quantitative polymerase chain reaction
RNA-seq	RNA sequencing
ROC	receiver operator characteristic
*RSAD2*	radical S-adenosyl methionine domain containing 2
SD	standard deviation
TCGA	The Cancer Genome Analysis
Viperin	virus inhibitory protein, endoplasmic reticulum-associated, interferon-inducible
WCC	white cell count
