# Supplementary material for: Clinical Implications of Upregulated RSAD2 Gene Expression in Hepatocellular Carcinoma
Source: Diseases. 2025 Dec 8;13(12):395. doi: 10.3390/diseases13120395 (PMC12731563; doi:10.3390/diseases13120395)
Supplement: Supplementary file 1 [file diseases-13-00395-s001.zip › diseases-3966006-supplementary.pdf]

Supplementary Table S1. Health-related quality of life characteristics of the patients.

	Continuous HRQoL factor						Dichotomized HRQoL factor[a]					
	Mean	SD	HR	95% CI		p-value	N <50	N ≥50	HR	95% CI		p-value
EORTC QLQ-C30												
Physical Functioning	73.78	23.46	0.374	0.294	0.476	<0.0001	55	254	2.145	1.585	2.904	<0.0001
Role Functioning	74.54	32.77	0.508	0.428	0.604	<0.0001	61	248	2.457	1.872	3.224	<0.0001
Emotional Functioning	71.17	23.56	0.714	0.543	0.938	0.0157	51	258	1.290	0.964	1.724	0.0862
Cognitive Function	77.45	23.31	0.654	0.505	0.846	0.0012	27	282	1.731	1.277	2.347	0.0004
Social Functioning	68.18	30.05	0.536	0.434	0.660	<0.0001	71	238	2.136	1.647	2.770	<0.0001
Global health status	53.43	25.11	0.428	0.329	0.555	<0.0001	105	204	1.785	1.389	2.293	<0.0001
Fatigue	42.57	29.70	2.248	1.839	2.748	<0.0001	189	120	2.418	1.879	3.111	<0.0001
Nausea and vomiting	12.41	22.13	1.754	1.370	2.246	<0.0001	274	35	2.111	1.454	3.066	<0.0001
Pain	30.58	30.61	2.042	1.680	2.482	<0.0001	217	92	2.178	1.673	2.835	<0.0001
Dyspnoea	29.56	31.38	1.739	1.431	2.114	<0.0001	234	75	1.960	1.488	2.581	<0.0001
Insomnia	43.91	35.98	1.481	1.245	1.761	<0.0001	177	132	1.660	1.296	2.125	<0.0001
Appetite loss	33.01	34.76	2.178	1.830	2.593	<0.0001	219	90	2.787	2.132	3.644	<0.0001
Diarrhoea	18.23	27.82	1.467	1.167	1.846	0.001	266	43	1.548	1.086	2.206	0.0157
Financial difficulties	51.56	36.96	1.285	1.086	1.521	0.0035	149	160	1.403	1.098	1.793	0.0068
C30 Index score	30.54	18.81	3.973	2.903	5.436	<0.0001	257	52	3.170	2.298	4.372	<0.0001
EORTC QLQ-HCC18												
Fatigue	33.62	24.91	2.451	1.924	3.122	<0.0001	240	69	2.330	1.751	3.100	<0.0001
Body Image	25.57	23.47	1.997	1.608	2.480	<0.0001	231	78	2.718	2.059	3.587	<0.0001
Jaundice	22.01	20.44	1.468	1.129	1.909	0.0042	259	50	1.421	1.030	1.960	0.0325
Nutritional concern	27.17	21.93	2.912	2.203	3.851	<0.0001	260	49	2.919	2.106	4.045	<0.0001
Pain	21.52	23.72	2.071	1.607	2.669	<0.0001	254	55	1.680	1.231	2.293	0.0011
Fever	5.72	13.75	1.745	1.127	2.701	0.0126	300	9	1.544	0.726	3.285	0.2592
Sex life	26.21	32.56	1.229	1.013	1.490	0.0364	240	69	1.525	1.145	2.032	0.0039
Abdominal swelling	28.91	33.95	2.008	1.673	2.409	<0.0001	229	80	2.955	2.239	3.901	<0.0001
HCC18 index score	23.84	16.49	3.702	2.653	5.167	<0.0001	281	28	2.498	1.657	3.765	<0.0001

a: For univariate cox proportional hazards analyses of dichotomized of HRQoL variables, worse (≥50) symptom scores were compared with better scores (<50); worse (<50) functioning and global health status scores were compared with better scores (≥50).

Legends: CI – confidence interval; EORTC – the European Organization for Research and Treatment; HR – hazard ratio; HRQoL – health related quality of life; SD – standard deviation .
